# Ring-stripping retrograde common carotid endarterectomy: case report

**DOI:** 10.1590/S1516-31802002000500007

**Published:** 2002-09-02

**Authors:** Eduardo Toledo de Aguiar, Alex Lederman, Patrícia Matsunaga

**Keywords:** Carotid artery, Arteries, Endarterectomy, Atherosclerosis, Cerebrovascular disease, Artéria carótida, Artérias, Aterosclerose, Endarterectomia, Doença cérebro-vascular

## Abstract

**CONTEXT:**

Total occlusion of the common carotid is rare and the indications and techniques for surgical treatment are still a matter of controversy.

**OBJECTIVE:**

To demonstrate the feasibility of retrograde common carotid endarterectomy.

**DESIGN:**

Retrospective case report study.

**SETTING:**

Tertiary care private hospital.

**PARTICIPANTS:**

Three patients underwent ring-stripping retrograde common carotid endarterectomy. Their ages were 81, 68 and 65 years. All were hypertensive with generalized atherosclerosis, two had diabetes mellitus, and one had undergone coronary artery bypass some years earlier and had non-dialytic chronic renal insufficiency. Symptoms of brain ischemia were present in two patients. All patients had total occlusion of the common carotid, extending from the origin to the bifurcation and localized in the right common carotid in two cases. In two cases the internal carotid artery was also occluded.

**MAIN MEASUREMENTS:**

Postoperative early mortality and stroke rate, and the medium and long-term endarterectomy patency.

**RESULTS:**

There were no deaths. One patient had a transient ischemic attack. All endarterectomies were patent after eight months, four years and seven years of follow-up.

**CONCLUSION:**

There is low mortality, and the procedure can be done through only one cervical incision. Tandem lesions of the carotid arteries can be treated together. It is suitable for long total occlusions of the common carotid, and long-term patency.

## RESUMO

**CONTEXTO:**

Obstruções da carótida comum são pouco freqüentes, e as indicações e técnicas de tratamento cirúrgico são motivo de controvérsia.

**OBJETIVO:**

Apresentar a aplicabilidade da endarterectomia retrógrada da carótida comum com anel.

**TIPO DE ESTUDO:**

Estudo retrospectivo de relato de casos.

**LOCAL:**

Hospital de cuidados terciários privado da cidade de São Paulo.

**PARTICIPANTES:**

Três doentes operados para endarterectomia retrógrada da carótida comum com anel. As idades foram 81, 68 e 65 anos, todos hipertensos com aterosclerose generalizada, dois diabéticos, um com antecedentes de revascularização do miocárdio e insuficiência renal não-dialítica. Dois apresentavam sintomas compatíveis com isquemia cerebral. As obstruções arteriais se estendiam da origem à bifurcação da carótida comum nos três casos, localizadas na carótida direita em dois casos e acompanhadas de obstrução total da carótida interna em dois casos.

**VARIÁVEIS ESTUDADAS:**

Mortalidade imediata, número de acidentes vasculares cerebrais isquêmicos imediatos e função da restauração arterial a médio e longo prazos.

**RESULTADOS:**

Não houve óbitos, um doente apresentou ataque isquêmico transitório no período pós-operatório imediato e todas as restaurações arteriais estão pérvias após segmentos de oito meses, quatro anos e sete anos.

**CONCLUSÃO:**

Mortalidade ébaixa, pode ser feito por meio de apenas uma incisão cervical, obstruções concomitantes da bifurcação e da carótida comum podem ser tratadas simultaneamente, pode ser empregada em oclusões totais longas da carótida comum e a durabilidade é longa.

**Figure 1:**
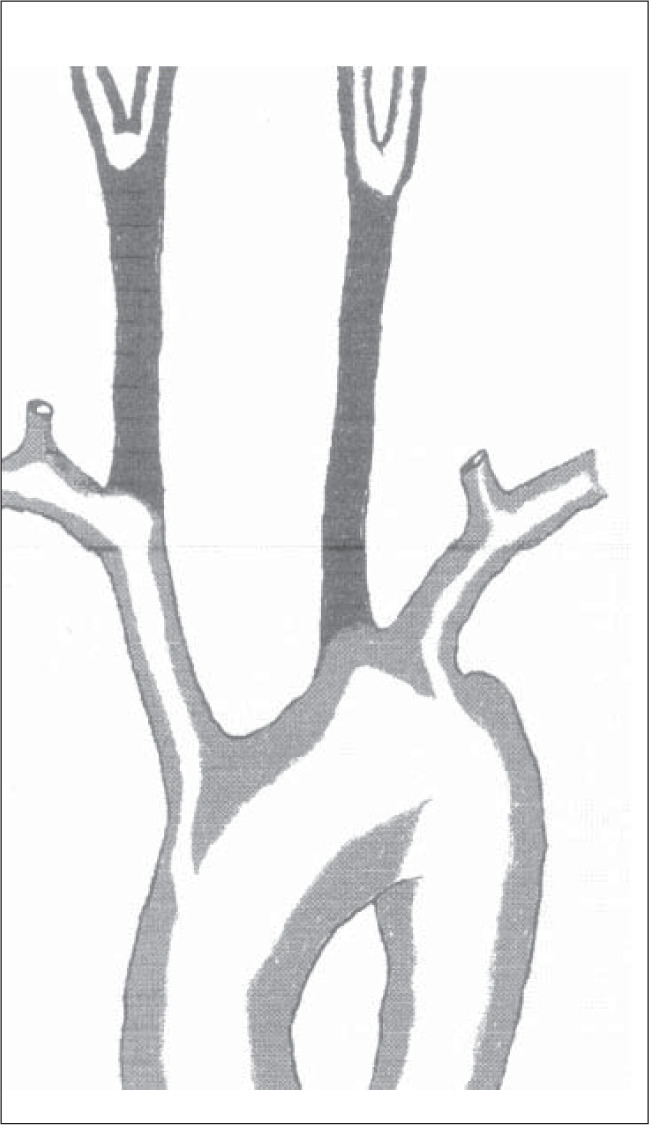
Site of common carotid obstruetions. Right eqmmon carotid - 2 cases. Left common carotid - 1 case.

Total occlusion of the common carotid artery is an important cause of brain ischemia, but it is not as frequent as carotid bifurcation stenosis. Moore et al. (1967) found it in 5% of their patients.^1^ In our own much smaller experience we have found similar rates: out of 65 patients operated on consecutively for cerebral ischemia, there were three with common carotid occlusion, representing 4.6% (Figure 1).^2^

Surgical treatment for occlusive disease of the supra-aortic trunk is not done very often. There is controversy about access: trans-thoracic or extra-thoracic and, in the last few decades, endovascular treatment has been preferred in certain cases.^3–5^

Our purpose was to report on three cases that demonstrate the feasibility of ring-stripping retrograde common carotid endarterectomy.

## CASE REPORT

**Case 1:** G.S.S., an 81-year-old female patient who was diabetic, hypertensive and a non-smoker, presented critical ischemia of the right lower limb with gangrene of the extremity of the foot. Popliteal and distal pulses were absent from both lower limbs. The right carotid pulse was absent and a bruit was heard at the level of the left carotid bifurcation. Arteriography revealed total obstruction of the right common carotid, but the right carotid bifurcation was not adequately seen. There was 60% stenosis at the left internal carotid origin and bilateral femorotibial obstruction with only the peroneal artery as a run-off on the right limb. Exploration of the right carotid bifurcation, common carotid endarterectomy and femoral-peroneal bypass were scheduled to be done at same time. The indication for exploration of the right carotid artery bifurcation was based on the premise that, if right internal carotid artery flow could be restored, the risk of brain ischemia after femorotibial reconstruction would be lower: there were no cerebral ischemic symptoms.

**Figure 2:**
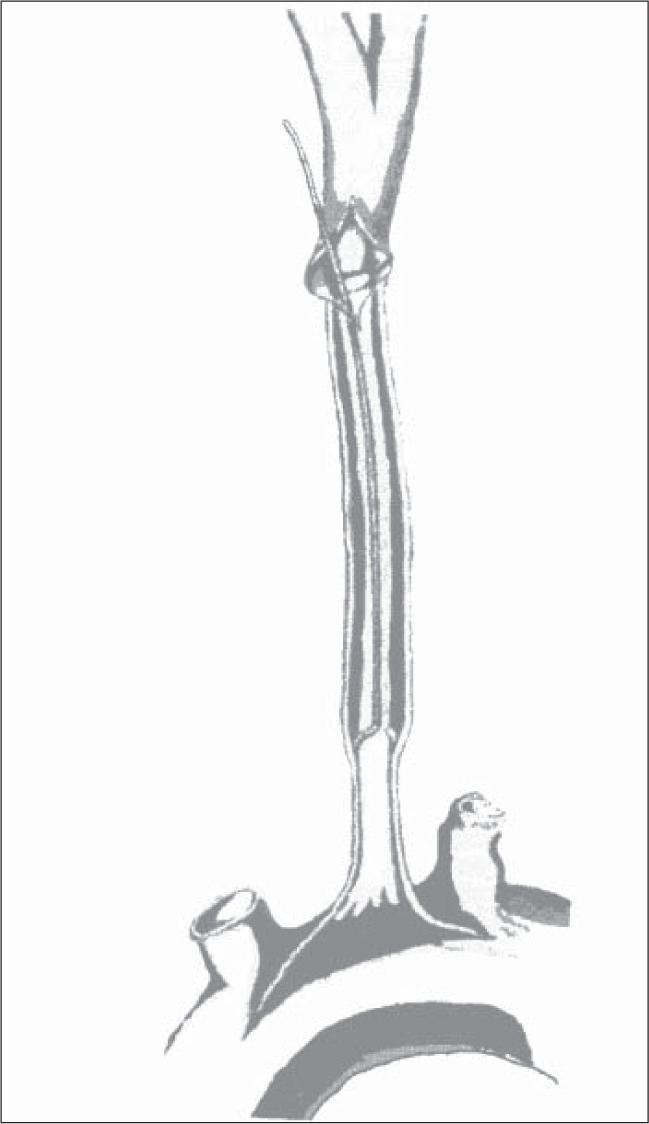
Ring-stripping retrograde common carotid endarterectomy technique.

On November 19, 1992, she underwent surgery. Surgical exploration of the carotid bifurcation revealed total occlusion of the internal and common carotid arteries. The endarterectomy of the external carotid origin was performed via an arteriotomy at the carotid bulb and, using the same cleavage plane, the ring-stripper was introduced back up through the common carotid artery until it reached the brachiocephalic trunk (Figure 2). At this point, resistance to the passage of the ring-stripper decreased abruptly, as the core was spilled out through the arteriotomy by means of the blood flow. The ring was introduced again to deal with any debris, and the arteriotomy was sutured. The pulse was restored to the external and common carotid arteries. This operation was followed by a femoral-peroneal bypass, but the saphenous vein was not long enough and a superficial femoral endarterectomy was done in association with the superficial femoral-peroneal bypass.

This restoration occluded on the following day and thigh amputation was performed eight days later. On the second postoperative day, the patient presented a transient ischemic attack, characterized by left hemiparesis that regressed totally. The patient refused left carotid endarterectomy after recovering, and was discharged from hospital. After a seven-year follow-up the patient is doing well and has not had any stroke or transient ischemic attack. A duplex-scan has shown some irregularities on the right common carotid artery, but without any significant stenosis.

**Case 2:** A.T.B., a 65-year-old male patient, was hypertensive, non-diabetic and a smoker. He was a businessman taken off his duties due to memory loss and disorientation. The physical examination revealed absence of the left temporal and common carotid pulses. All other pulses were normal. There was bruit at the level of the right carotid bifurcation. Computerized tomography of the brain revealed multiple ischemic lesions in both hemispheres and arteriography showed total occlusion of the left common carotid artery and 80% stenosis of the right internal carotid artery. The left carotid bifurcation was not adequately seen.

He was operated on November 17, 1993. Exploration of the left carotid bifurcation revealed totally occluded internal and common carotid arteries and severe stenosis of the left external carotid artery origin. As described above, the arteriotomy at the carotid bulb allowed endarterectomy of the external carotid artery to be performed, and the introduction of the ring-stripper back up through the common carotid until the resistance decreased and the core spilled out. After suturing the arteriotomy, pulsatile blood flow was restored in the left common and external carotid arteries.

One week later, right carotid bifurcation endarterectomy was done in the normal manner. The patient was discharged from hospital on the 5^th^ postoperative day after this second operation with no complications. He was followed up for four years, until April 1997, by which time he had partially returned to his professional activities. The duplex-scan that was performed showed some irregularities on both carotid arteries, but with no significant stenosis.

**Case 3:** A.J.D. was a 68-year-old diabetic and hypertensive patient, who eight years earlier had undergone a coronary artery bypass. At that time, he quit his smoking habit and had manifestations of leprosy, which were cured. From that time onwards, he suffered from non-dialytic renal insufficiency. The symptoms reported were one episode of left hemiparesis that lasted for 20 minutes and disappeared completely one-and-a-half months before coming to us, and an episode of loss of consciousness that led him to a car crash 15 days before coming to us. He also complained of right-eye vision blurring. The temporal and right carotid pulses were not palpable, nor were the dorsalis pedis and posterior tibial on the right lower limb. An ischemic lesion in the right brain hemisphere was detected by computerized tomography. A duplex-scan revealed obstruction of the right common carotid, with pervious internal and external carotid arteries. Angiotomography confirmed the duplex-scan findings.

On February 25, 2001, the patient underwent retrograde common carotid endarterectomy using the ring-stripper. After exploration of the right carotid bifurcation, the internal and external carotid arteries were found to be patent. The common carotid artery was totally occluded and there was an atheroma plaque at the internal carotid artery origin. Endarterectomy of the carotid bifurcation and common carotid artery was performed as described earlier.

The patient was discharged from hospital after 24 hours with no complications. All symptoms have disappeared except for a sensation of dizziness that occurs episodically. Control duplex-scans have revealed full-length dilatation (1.5 cm) of the common carotid artery and 50% to 70% stenosis at its origin. The patient continues under observation.

## DISCUSSION

Obstructive disease of the common carotid artery is not often seen. Its incidence varies from 1% to 5%.^6^ Controversy still remains with regard to the indications and techniques for surgical treatment.

The symptoms of brain ischemia may be caused by two factors: hemodynamic changes and embolism. Hemodynamic factors may be dominant, with brain and eye hypoperfusion being responsible for transient ischemic attacks or infarcts in both the carotid and vertebrobasilar regions. Embolic events may occur even when there is total common carotid occlusion.

Some surgeons believe that occlusions originate at the end of thrombosed segments of common carotid artery at the carotid bifurcation or at the end of the internal carotid artery at the circle of Willis.^7^ Moore et al. (1967) stated that total occlusion of the common carotid artery originates from atheroma plaque located at one of the following four points: 1. the origin of the common carotid; 2. the innominate artery or aortic arch; 3. the middle segment of the common carotid (between the origin and the bifurcation); or 4. immediately before the bifurcation and origin of the internal and external carotid arteries. Moore et al. (1967) also found the most frequent lesions provoking total occlusion of the common carotid to be those located at the bifurcation, with thrombosis progressing as far as the common carotid origin at the in-nominate artery or aortic arch.

Atheroma plaques located at the origin of the common carotid artery rarely progress to total occlusion.^1^ Frequently, the atherosclerotic lesions on supra-aortic trunks are multiple. Vogt et al. (1982) studied brachiocephalic arterial reconstruction in 97 patients and found that 57 of them had multiple stenoses greater than 50%.^8^ The extent and multiple locations of atheroma plaques and obstructions cause the symptoms to be varied and related both to the carotid and vertebro-basilar regions. In such patients, atherosclerotic disease is very often generalized, with coronary artery disease found in 45% and peripheral arterial obstructive disease (PAOD) in 27%. Hypertension is also present in 50% of such patients.^2,7^

All the patients presented here were hypertensive and had diffuse atherosclerotic disease compromising the coronary and peripheral arteries. One patient had diabetes mellitus. All had atheroma plaques at the carotid bifurcation, in continuity with total occlusion of the common carotid artery. Typical symptoms of extracranial arterial obstructive disease occurred in case 3, and the ischemic brain lesion uncovered by the CT scan was ipsilateral to common carotid obstruction. The patient in case 2 presented symptoms of dementia, but the CT scan revealed multiple ischemic lesions in both brain hemispheres, and so it is possible that the symptoms were ischemic in origin, especially because of the patient's improvement after surgical therapy. In case 1, the indication was prevention of stroke in a patient undergoing major surgery: this is absolutely a doubtful indication. Restorative surgery of supra-aortic trunks has been indicated in symptomatic patients.^10–12^

Vessel patency distal to arterial occlusion (runoff vessels) is important for surgical planning. Arteriography of aortic arch catheterization sometimes fails to demonstrate runoff. In earlier days, the patency of internal or external carotid arteries or both was only revealed upon surgical exploration, as happened in cases 1 and 2. More recently, colored duplex-scan and angiotomography have enabled surgeons to identify distal runoff, as happened in case 3.^8^

Total occlusion of the common carotid artery is frequently associated with internal carotid occlusion, leaving only the external carotid artery patent. The importance of this artery as the origin of collateral circulation to the brain has already been emphasized.^13–15^ AbuRhama et al. (1998), when studying the natural history of total occlusion of the bilateral internal carotid in a group of patients, showed that after surgical brain revascularization patients presented long-term mortality and stroke rates that were lower than for those treated conservatively. Most of them had their blood flow restored through the external carotid artery.^16^

The three patients presented here illustrate this fact quite well. Two of them only had the external carotid artery as a distal runoff. Lamberth (1983) demonstrated the importance of restoring blood flow to the external carotid artery, especially when there are multiple supra-aortic trunk obstructions.^17^

Many techniques for surgical treatment of obstructive disease of the common carotid have been proposed. The mortality resulting from aorto-carotid bypasses done through sternotomy or thoracotomy used to be as high as 20%, but has fallen to 6% or less nowadays.^3,18^ Thirty-four patients were operated on consecutively for aorto-carotid and/or subclavian bypasses with no mortality, between 1985 and 1990, in the Vascular Surgery Service of the University of São Paulo Medical School's Clinical Hospital.^19^ Although mortality has fallen remarkably, the transthoracic repair of supra-aortic arteries is followed by high morbidity related to thoracic drainage and the multiple cervical incisions needed to complete the procedure, which implies a long postoperative stay in hospital. Extra-thoracic reconstruction has led to lower mortality and morbidity. Its high long-term patency rate and capacity for alleviating symptoms has made subclavian-carotid bypass the treatment of choice for common carotid obstructions.^4,20^

But even with these good results, a problem still remains: the multiple incisions needed for performing this bypass. Endovascular therapy offers the opportunity to treat such patients without any incision. An early success rate of 92.3% has been reported among these patients, and there are surgeons who are combining carotid bifurcation endarterectomy with intraoperative dilation of proximal common carotid stenosis to treat these lesions in tandem.^9,21^ The endovascular technique has a limitation: not infrequently, the guide wire will not cross stenosing or total occlusive lesions, especially when these are long and compromise the whole length of the common carotid, as in the cases described here.

Retrograde ring-stripping common carotid endarterectomy, as introduced by Moore, Blaisdell and Hall in 1967 and described in this paper, is an alternative for cases similar to those reported here, i.e. atherosclerotic obstruction of the full length of the common carotid artery. It is important for surgeons to practice ring endarterectomies in other regions like the abdominal aorta or the femoral or iliac arteries, so that they know exactly what resistance is offered by subintimal tissue.^22^

There are few reports dealing with this technique, and we were only able to find 14 retrograde endarterectomies performed as described here by different surgeons. There were two deaths: one case in which saline endarterectomy was used to treat a long internal carotid artery occlusion in addition to a common carotid retrograde endarterectomy, and another patient who died two weeks after the operation because of congestive heart failure.^8,12,18^ Some surgeons believe the risk of perforation or aortic dissection is high and that this technique should be banished. This does not seem to be the case, but stenosing residual plaques in the proximal common carotid have been reported, as happened in case 3.^11^ Moore et al. (1967) stated that this lesion rarely progresses to total obstruction.^1^ But it may be a source of emboli, although this has not often been reported in the literature.

In conclusion, with the results presented here and those from other services, it may be taken that the procedure can be done through only one cervical incision, and tandem lesions of the carotid arteries can be treated together. It is suitable for long total occlusions of the common carotid, and long-term patency is maintained. Bearing in mind the limitations imposed by the small sample size, it is thought that mortality is low.
